# All You Need to Know about the Kinetics of Thermally Stimulated Reactions Occurring on Cooling

**DOI:** 10.3390/molecules24101918

**Published:** 2019-05-18

**Authors:** Tatsiana Liavitskaya, Sergey Vyazovkin

**Affiliations:** Department of Chemistry, University of Alabama at Birmingham, 901 S. 14th Street, Birmingham, AL 35294, USA; tliavi@uab.edu

**Keywords:** activation energy, Arrhenius equation, cooling, crosslinking, decomposition, isoconversional method, model-free kinetics, rate constant

## Abstract

In this tutorial overview article the authors share their original experience in studying the kinetics of thermally stimulated reactions under the conditions of continuous cooling. It is stressed that the kinetics measured on heating is similar to that measured on cooling only for single-step reactions. For multi-step reactions the respective kinetics can differ dramatically. The application of an isoconversional method to thermogravimetry (TGA) or differential scanning calorimetry (DSC) data allows one to recognize multi-step kinetics in the form of the activation energy that varies with conversion. Authors’ argument is supported by theoretical considerations as well as by experimental examples that include the reactions of thermal decomposition and crosslinking polymerization (curing). The observed differences in the kinetics measured on heating and cooling ultimately manifest themselves in the Arrhenius plots of the opposite curvatures, which means that the heating kinetics cannot be used to predict the kinetics on cooling. The article provides important background knowledge necessary for conducting successful kinetic studies on cooling. It includes a practical advice on optimizing the parameters of cooling experiments as well as on proper usage of kinetic methods for analysis of obtained data.

## 1. Introduction

This article summarizes our experience in studying the kinetics of thermally stimulated reactions taking place during continuous cooling. We have studied the kinetics of thermal decomposition and thermal polymerization by using two methods of thermal analysis: differential scanning calorimetry (DSC) and thermogravimetric analysis (TGA). Note, DSC and TGA are used broadly for kinetic studies of these types of reactions [1,2,3]. However, these studies are conducted routinely on heating, i.e., by continuously raising temperature. The use of a continuous heating program is primarily the matter of convenience. As long as a reaction is initiated thermally it is convenient to perform it by gradually adding heat. The energy gained is then converted into continuously intensified translational motion of molecules and vibrational motion of chemical bonds. At some point the bonds starts breaking producing reactive species that initiate the process. Ultimately, continuous heating drives the reaction to completion. The data obtained in a continuous heating run can then be used to gain some insights into the reaction mechanism as well as to develop a kinetic model suitable for research and industrial applications. 

We have strived to accomplish the same tasks but using continuous cooling runs. Note that cooling segments have been employed in kinetic studies when using the techniques of temperature modulated DSC and TGA as well as of controlled rate thermal analysis. However, in these techniques the cooling segments are invariably combined with heating segments. When applied to thermally stimulated reactions these techniques do not employ an overall cooling program, i.e., the temperature at completion of a reaction is not lower than at its initiation. Rather, the temperature at completion is either higher (overall heating program) or the same (quasi-isothermal program) as the temperature at initiation. In our studies, the kinetics has been measured entirely under continuous cooling, i.e., when temperature decreases progressively throughout the reaction progress. Our research was motivated by both scholastic and pragmatic interest described in the following sections. When we initiated this research we had ventured in truly uncharted territory. Suffice to say that when we started we simply had troubles to detect the DSC signal on cooling for a reaction that had been well studied on heating. Over time, we have learned how to obtain reliable kinetic data, perform kinetic computations, and interpret the obtained results. This is the experience that we share in the present article. Its objective is to create a single reference that covers all basics needed for one to successfully conduct a kinetic study of thermally stimulated reactions occurring on continuous cooling. To accomplish this objective, we discuss scholastic and pragmatic motivation, the theory and praxis of measurements, kinetic computations, and representative examples. 

## 2. Scholastic Motivation

As already stated, DSC and TGA are routinely used to measure the kinetics of thermally stimulated reaction on heating. However, obtaining the results exclusively on heating unavoidably gives rise to a limited picture of the process. Consider a mathematical function of two independent variables *x* and *y*, e.g., f(*x*,*y*) = m*x* + n*y*, with positive values of m and n. Simultaneously increasing both variables results in increasing f(*x*,*y*) that makes it difficult to explore the individual effects of *x* and *y* because the effects are similar. On the other hand, when the variables are changed in the opposite ways, i.e., one increases and another decreases, they have distinctly different effects on f(*x*,*y*) that affords the possibility of better understanding of the individual effects. Respectively, an analogy can be drawn with thermally stimulated reactions whose rate depends on conversion, *α* and temperature, *T*. Carrying out a reaction on continuous heating and cooling allows one to vary *T* in opposite directions and, thus, to learn more about the individual effects of both variables, i.e., to obtain a more complete kinetic picture of the process.

Although any reaction can be performed on cooling, the most interesting results should be expected for multi-step reactions. This is because for multi-step reactions the effective activation energy typically depends on both temperature and conversion [4,5]. In this situation, one can expect different results for heating and cooling. This can be illustrated by a simplistic algebraic example. Consider a hypothetical reaction that takes place in the temperature region from 300 to 400. To keep things as simple as possible we will use dimensionless temperature. Let us introduce the *α* vs *T* functions that change from 0 to 1 in that temperature range on heating:

(1) $$\alpha^{+}=0.01T-3$$

and on cooling:

(2) $$\alpha^{-}=-0.01T+4$$

Let us now introduce a dimensionless activation energy that depends on both *α* and *T* in some very simple form:

(3) E=α·T

Now we can obtain temperature (Equations (4) and (5)) and conversion (Equations (6) and (7)) dependences for the activation energy on heating and cooling by rearranging and substituting Equations (1) and (2) into the activation energy expression, Equation (3). The results of these manipulations are shown below in Equations (4)–(7):

(4) $$E^{+}\left(T\right)=0.01T^{2}-3T$$

(5) $$E^{-}\left(T\right)=-0.01T^{2}+4T$$

(6) $$E^{+}\left(\alpha \right)=100\alpha^{2}+300\alpha$$

(7) $$E^{-}\left(\alpha \right)=-100\alpha^{2}+400$$

**Figure 1:**
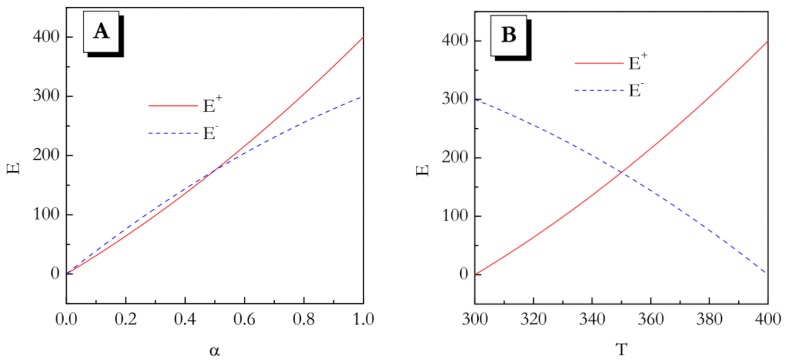
Schematic presentation of the E vs α (**A**) and E vs T (**B**) dependences for heating and cooling based on Equations (1)–(7).

These dependences are illustrated graphically in Figure 1A,B. Even though from the *E* vs *α* trends it seems that the values do not differ significantly, especially for *α* < 0.5, the temperature dependences of the activation energy demonstrate opposite trends. This means that in the heating experiment the activation energy increases with increasing temperature, whereas on cooling it decreases with increasing temperature. 

Naturally, the activation energy represents the slope of the Arrhenius plot:

(8) $$\ln k=\ln A-\frac{E}{RT}$$

where *k* is the rate constant, and *A* is the preexponential factor. The opposite directions of a change in *E* with increasing *T* means that the Arrhenius plots have to have the opposite curvatures. That is, if on heating the plot is concave up (*E* increase with *T*), on cooling it would be concave down (*E* decreases with *T*). Therefore, the heating and cooling kinetics cannot be reduced to each other. The major conclusion of this simplistic example is that for the multi-step reactions whose activation energy depends on both temperature and conversion, the kinetic parameters evaluated on heating may not suitable for predicting the kinetic behavior on cooling. 

If a reaction is single-step, its activation energy is constant, i.e., independent of either α or T. Then, in line with the aforementioned arguments, the kinetics on heating and cooling should be the same. In this case, the kinetic parameters evaluated on heating would be suitable for predicting the kinetic behavior on cooling. 

The considerations presented in this section allow one to generate the following central hypothesis for kinetic studies on continuous cooling. For a single-step reaction the kinetics on heating and cooling should be practically identical, whereas the respective kinetics should differ significantly for multi-step processes. Of course, these two types of processes are easy to identify by employing an isoconversional method to evaluate the activation energy as a function of conversion. 

## 3. Pragmatic Motivation

The existence and importance of reactions occurring on cooling has been recognized in the literature. The issue has been discussed in connection with decomposition of amino acids [6], pyrolysis of hydrocarbons [7], cracking of heavy oils [8], pyrolysis of heterogeneous materials such as almond shells, municipal solid waste, lignin, and polyethylene [9]. It has also been brought up in regard to epoxy materials crosslinking [10,11] and vulcanization of rubber [12,13,14,15]. That is, there is no doubt that cooling is an integral part of many manufacturing processes and, as such, it affects the properties of the final product. Therefore, studying the kinetics of thermally stimulated reactions on cooling is not only of scholastic but also of practical interest. 

In the absence of such studies, it is often assumed that the reaction rate is negligibly small during cooling or that kinetics determined on heating can be applied to the cooling conditions [10,16,17]. However, as demonstrated in the previous section, the similarity of the kinetics on heating and cooling should be expected only for single-step reactions. For multi-step reactions, the respective kinetics are likely to be different that means that one has to study the kinetic on cooling in order to understand the processes that involve cooling segments. Despite the obvious need, there had been no systematic studies of thermally stimulated reactions on cooling until our initiatory work [18]. A likely reason for this situation is that performing kinetic measurements on cooling is quite challenging. The issue is discussed in the next two sections.

## 4. Theory of Measurements on Cooling

**Figure 2:**
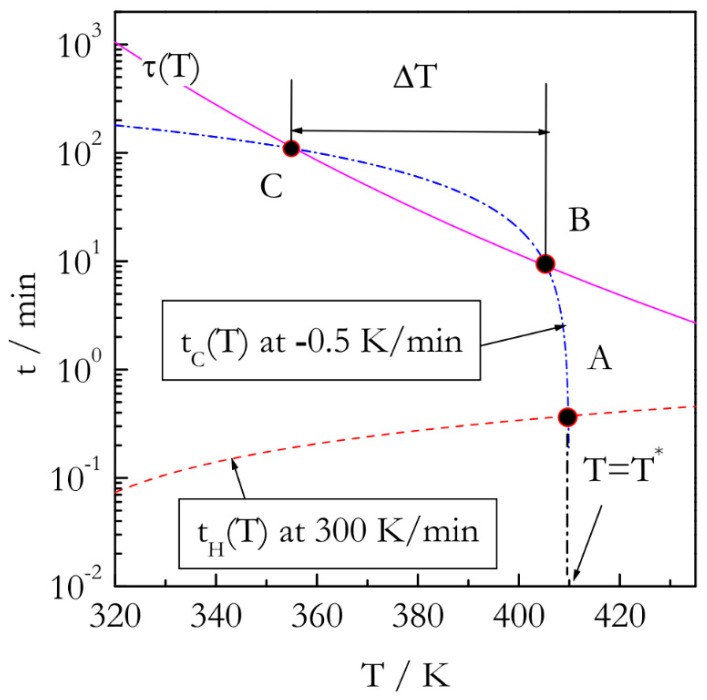
Temperature dependence of characteristic time, τ(T) (solid line) for a reaction with E = 60 kJ mol^−1^ and A = 10^5^ s^−1^. Turning temperature, T* = 410 K. Dash line: heating time, dash-dot line: cooling time. Time is on log scale.

The major problem with performing kinetic measurements on cooling is that the respective experiments have to satisfy two opposing conditions. First, cooling has to be initiated from the temperature, T*, at which a reaction proceeds rapidly enough to follow its rate reliably. Second, this temperature has to be reached so that the reaction cannot proceed to any significant extent. The idea of satisfying both conditions experimentally is presented in Figure 2. First off, it is necessary to outrun the reaction while heating. It means that the temperature needs to be raised over a period of time that is markedly shorter than the characteristic reaction time, *τ*(*T*). One can define *τ*(*T*) via the reciprocal rate constant as [19]:

(9) $$\tau \left(T\right)=k^{-1}={\left[A\exp\left(\frac{-E}{RT}\right)\right]}^{-1}$$

To make our estimates more realistic we take the values of A and E as 10^5^ s^−1^ and 60 kJ mol^−1^ that would be characteristic of a process such as polymerization. If the reaction temperature is raised at constant rate *β_H_*, the temperature, T* will be reached at the following time:

(10) $$t_{H}\left(T^{*}\right)=\frac{T^{*}-T_{0}}{\beta_{H}}$$

where *T*_0_ is the starting, e.g., ambient, temperature, and the subscript *H* denotes heating. Outrunning the reaction on heating means satisfying the condition *t_H_*(T*) << *τ*(T*). For instance (Figure 2), if the reactant is heated rapidly, e.g., at 300 K min^−1^ to T* = 410K (point A), the heating time *t_H_*(T*) would be close to 0.4 min. The characteristic reaction time at this temperature is roughly 8 min. That is, the reaction would not have time to proceed to any significant extent before reaching T*. Once T* is reached, we start cooling the reactant slowly, e.g., at *β_C_* = −0.5 K min^−1^. The respective cooling time is then determined as:

(11) $$t_{C}\left(T\right)=\frac{T-T^{*}}{\beta_{C}}$$

Since |*β_C_*| << *β_H_*, the time scale of the cooling run will soon become similar to the time scale of the characteristic reaction time, which can be expressed as *t_C_*(*T*) ~ *τ*(*T*) (temperature drops below that at point B in Figure 2). The conditions of the similar time scales would hold until decreasing temperature slows down the reaction so that characteristic reaction time becomes longer than the time scale of cooling (temperature drops below that at point C in Figure 2). Then, the reaction would cease to be measurable. The region between points B and C, would provide a temperature window, Δ*T*, within which the reaction kinetics can be measured during continuous cooling. 

As follows from the above discussion, successful measurements of the reaction kinetics on cooling are contingent on an appropriate selection of the experimental parameters *β_H_*, *β_C_*, and T*. Basic principles of selecting and optimizing these parameters are discussed in the next section.

## 5. Praxis of Measurements on Cooling

As discussed above, the experiments on cooling have to start at elevated temperature and complete while the system is cooling down. In DSC or TGA this is done by rapidly heating a reagent to an elevated temperature, T*, where the reaction rate is sufficiently fast. Once this temperature is reached the fast heating is switched to a slow cooling. For a limited number of reactions with a very long induction period such as decomposition of NiC_2_O_4_ [20] this procedure can be modified. The instrument can first be preheated to T*. Then, the reagent can be introduced into the preheated furnace for a short isothermal hold to stabilize the temperature followed by a cooling segment. However, this works only when a reaction does not start immediately, i.e. has a significant induction period. If a reaction has no induction period, opening and closing the furnace will cause significant disturbance in the experimental data at the beginning of the reaction and these would not be suitable for kinetic analysis. 

The majority of reactions do not have a long induction period, thus, a fast heating segment is necessary. In a regular DSC or TGA, the fastest available heating rate is about hundreds of degrees per minute. Such heating rates are entirely sufficient to minimize the reaction progress during heating. However, the use of excessively fast heating rates may introduce unnecessary disturbance into the measured signal. When the instrument switches from fast heating to slow cooling, it continues to heat up overshooting the set turning temperature. Overshooting is larger for faster heating rates in general and for the TGA measurements in particular due to slower response time of the latter. Generally, for the DSC experiments when using the heating rate of 100 °C min^−1^, overshooting is not much larger than 5 °C, and the instrument catches up with the cooling program within ~10 s. As for the TGA measurements, overshooting is much larger and the adjustment time is longer. Thus, the disturbance in the measured signal results from the fact that when one temperature program is switched to another one, the instrument unavoidably needs some time to adjust to the new temperature program. This effect is especially strong when switching between heating and cooling. Also, the theory of the kinetic measurements on cooling requires switching from a fast heating rate to a slow cooling rate that additionally prolongs the adjustment time. Nevertheless, the overall disturbance in the instrumental signal can be diminished by properly choosing experimental parameters of the experiments. 

The choice of the heating rate depends on the instrument used and nature of the process studied. For instance, for reactions with sigmoid or accelerating types of kinetics, the heating rate in DSC runs can be as slow as 30 °C min^−1^ [21]. For this type of kinetics the rate is slowest when the reaction starts. This means two things. First, such kinetics are easy to outrun on heating. Second, the signal disturbance caused by switching from heating to cooling does not have a significant effect on the data collected during the cooling segment. The longer the induction period is, the slower the heating rate can be. For instance, for decomposition of NiC_2_O_4_ a rather slow heating rate of 5 °C min^−1^ has been adequate for obtaining reliable data on cooling. 

It is significantly more difficult to deal with reactions that follow decelerating kinetics. This type of kinetics demonstrates the fastest rate at the beginning of a reaction. These reactions are more difficult to outrun on heating. Also, the signal disturbance caused by switching from heating to cooling may have a rather strong effect on the data measured during cooling. Thus, to diminish the detrimental impact of the signal disturbance the heating rate in the respective DSC measurements should be at least 100 °C min^−1^ [21].

For TGA measurements, the situation is more complicated. As discussed above, when a fast heating is switched to a slow cooling, there is a stabilization period during which the instrument adjusts to the new temperature program. The length of the stabilization period is associated with the instrument response time or the signal time constant. The larger the signal time constant is, the longer it takes to stabilize the signal after switching the program. For instance, in the TGA and DSC instruments used for our measurements on cooling [18,20,21,22,23], the signal time constants were 14 and 1.7 s, respectively. Thus, TGA stabilizes much slower. Consequently, the use of TGA imposes additional limitations on the choice of the heating rates. Thus, even for the processes with decelerating type of kinetics, the heating rate should not exceed 20 °C min^−1^. Because of the slow instrumental response time the fast heating program cannot be switched immediately to the slow cooling. Instead, TGA continues to heat up the system for some time, reaching temperatures significantly larger than T*. This, in turn, results in non-negligible reaction progress attained before the slow cooling program is established. Obviously, the faster the heating rate is, the more temperature overshoots above T*. At the same time, the use of excessively slow heating rate leads to a significant reaction progress during heating that, as a consequence, introduces larger error in the kinetic parameters estimated on cooling. In our experience the heating rates around 15–20 °C min^−1^ seem optimal.

The second experimental parameter that must be controlled is the turning temperature, T*. It determines how fast the reaction will proceed when the cooling segment starts. The higher the turning temperature is, the faster the reaction is and, as a result, the measurements on cooling will require the use of faster heating and cooling rates. Faster heating rate is required to minimize the reaction progress during the initial heating. Higher turning temperature also requires the use of faster cooling rates. This is because higher T* means that reaction rate is faster and that the process will be complete within a narrower temperature range during cooling. The use of narrow temperature ranges is generally undesirable because it leads to a greater uncertainty in evaluating the kinetic parameters. To expand the temperature range, one has to use faster cooling rates. As a rule of thumb, for autocatalytic (sigmoid) kinetics the turning temperature should be chosen as a DSC peak temperature of a heating run at 5 °C min^−1^. For the decelerating kinetics, T* should be selected ~10 °C higher. This is because on cooling the process decelerates due to decrease in temperature and decelerating reaction model. Faster deceleration leads to incomplete reaction on cooling and inability to evaluate kinetic parameters for higher extents of conversion. 

The aforementioned recommendations about choosing the turning temperature are mostly relevant to the measurements made by DSC that has a shorter response time and, thus, better control over the temperature program. Due to slower response time of TGA, the respective runs on cooling should generally be performed to keep the reaction rate slower. Therefore, the turning temperatures in the TGA runs should be kept about 10℃ lower than in the DSC runs. For instance, decomposition of Li_2_SO_4_∙H_2_O on cooling has been studied by using both experimental techniques, DSC and TGA [18,20,21]. In the DSC runs, the turning temperatures have been selected to be 100 and 110 °C whereas in the TGA measurements T* has been 95 °C [18,20]. As discussed before, the use of slower heating rates is required in the TGA measurements to minimize overheating. For example, for decomposition of Li_2_SO_4_∙H_2_O the heating rate has been 15 °C min^−1^ [20]. At the same time, such a low heating rate will unavoidably lead to partial reaction progress during heating. Thus, lower T* is chosen to reasonably minimize the effect of the partial reaction progress during heating on the overall kinetics on cooling. However, it should be kept in mind that the turning temperature cannot be lowered significantly. Low T* can lead to incomplete reaction on cooling that would make it impossible to evaluate the kinetic parameters reliably within the whole range of conversions. 

The last experimental parameter that needs to be optimized is the cooling rate. As mentioned before, the cooling rates need to be much slower than the heating ones. They are selected to be slower to ensure that the reaction reaches its completion during the cooling segment. For the DSC measurements, the cooling rates are typically chosen in the range of 0.1 to 2 °C min^−1^. For the slower cooling rate, 0.1 °C min^−1^ the reaction typically happens within a couple of degrees whereas for the faster one it may cover 20–40 °C range. A wider temperature range covered on cooling allows for evaluating kinetic parameters with a smaller uncertainty. 

In the measurements on cooling, the reaction continuously decelerates due to decrease in temperature. Thus, if the heating rate and the turning temperature are selected properly, the use of slower cooling rates typically leads to reaching 100% of conversion whereas for the faster ones it can be significantly smaller. It is not recommended to use the fast rates that lead to less than 90% conversion. This applies to both TGA and DSC measurements. However, as discussed earlier, the TGA should generally be performed under the conditions that maintain slower reaction rate. As a consequence, the fastest cooling rates in TGA should be slower than in DSC, typically not faster than 1 °C min^−1^. This enables the reaction system to stay longer at higher temperature and, thus, to attain full or nearly full conversion. 

To obtain adequate results for the kinetics on cooling, one should perform measurements with at least five different cooling rates. For the TGA experiments where cooling temperature region can be even narrower, more cooling rates may be required. It is noteworthy that the abovementioned recommendations on selecting the experimental parameters are not a set of strict rules. Rather, they are general directions on how to get a handle on controlling kinetic measurements on cooling. Each particular process may require a somewhat different experimental setup to achieve optimal condition of the measurements. It should also be remembered that all the experimental parameters are interconnected. If one parameter is changed, the other should also be readjusted. For instance, if the turning temperature is increased, the heating rate should be increased as well to minimize the reaction progress during heating. At the same time, the faster cooling rate can also be increased providing a wider temperature range for the cooling measurements.

Another important feature of the experiments on cooling is that they require knowing the final extent of the reactant conversion. In the heating measurements, the reaction normally attains complete conversion because the system is continuously heated promoting the reaction. On cooling, temperature decreases and so does the reaction rate. At some point, the temperature may become so low that process virtually ceases before reaching 100% conversion. Another reason why the reaction can stop is specific to polymerization reactions and associated with vitrification [22,23]. Our simulations have demonstrated that in the case of incomplete reactions one has to use the absolute values of conversion in order to retrieve correct kinetic parameters from the data [18]. For example, if at the fastest cooling rate the ultimate extent of conversion reached is 60%, for performing isoconversional calculations one has to use only the portion limited to 60% conversion from the data obtained at slower cooling rates. 

For decomposition reactions or any processes that involve a mass change, determination of the final extent of conversion is relatively straightforward. For both DSC and TGA data, it is done by measuring the mass before and after the run and comparing their ratio to the one experimentally evaluated on heating. This is especially important when the ultimate mass loss cannot be evaluated theoretically from the stoichiometry as in degradation of polymers. As for the stoichiometric reactions such as dehydration of hydrates one can use a theoretically evaluated mass loss for evaluating the complete extent of conversion. In the TGA runs, the mass is monitored continuously. Thus, it is possible to accurately detect partial reaction progress during heating. Therefore, to account for the related reaction progress and more accurately evaluate the conversion, it is recommended not to use the mass at the turning temperature. It is more appropriate to use the mass at the beginning of detectable decomposition that may start on heating. 

The kinetics of the processes that do not involve change in mass (e.g., polymerization) can be studied by DSC. In this case, determination of the extent of conversion relies only on measuring the reaction heat. This creates another challenge in the measurements on cooling. Switching the temperature programs at the turning temperature, causes a disturbance in the DSC signal. As a result, one cannot use regular DSC software to subtract the baseline because the baseline in the vicinity of the turning temperature is highly uncertain. This issue arises for any reaction. However, for the reactions that involve a change in the mass, the final extent of conversion can be established by weighing the sample before and after the experiment. Difficulty in subtracting the baseline can be overcome by using software such as MS Excel, and performing this procedure manually. By conducting multiple DSC runs with an empty pan, we have established that switching from fast heating to slow cooling causes a heat flow perturbation that decays in a manner similar to that defined by the Kohlrausch, Williams, and Watts (KWW) function [24,25]:

(12) $$y=1-\exp\left[-{\left(\frac{t}{\tau_{\mathrm{ef}}}\right)}^{\gamma }\right]$$

where *γ* is the stretch exponent, *t* is the time, and *τ*_ef_ is the effective relaxation time. This equation has been transformed to fit the baseline by taking *y* as (HF_0_ − HF_t_)/(HF_0_ − HF_f_). This transformation gives rise to the following equation [18]:

(13) $$HF_{t}=HF_{f}+\exp\left[-{\left(\frac{T^{*}-T}{T_{\mathrm{ef}}}\right)}^{\gamma }\right]\left(HF_{f}-HF_{0}\right)$$

where *HF*_t_ is the current heat flow, *HF*_0_ and *HF*_f_ are the heat flow values at the beginning and end of the reaction, T* is the turning temperature, and *T*_ef_ and *γ* are adjustable parameters. The parameters of the fit need to be chosen to satisfy the following criteria. First, the reaction should start as close as possible to the turning temperature and end where the DSC signal merges into the baseline value, i.e. when the process stops producing the latent heat. Second, the enthalpy of the reaction on cooling determined after subtracting the baseline should be consistent with the value determined in the heating runs. As mentioned before, for decomposition reactions, the thermal effect can be additionally verified by weighing the sample before and after the experiment. As for the processes that do not involve a mass change, it has been determined that the enthalpy of the reaction stays reasonably constant for one cooling rate and does not change much if the fitting parameters are somewhat varied. One also should rely on the value of the thermal effect obtained on heating as a reference value of the enthalpy for the measurements on cooling. It is noteworthy that even for the regular heating DSC measurements the choice of the baseline implies certain variability. Nevertheless, it is recommended to first practice to subtract a baseline in the cooling runs for the processes with a single-step kinetics where the activation energy is expected to be the same on heating and cooling and then move to more complicated reactions. 

## 6. Kinetic Computations for Cooling Data

All our kinetic computations on the cooling data were performed based on the recommendations of the ICTAC Kinetics Committee [26]. The effective activation energy, *E_α_*, was evaluated as a function of conversion with the help of an advanced isoconversional method [27] that is suitable for kinetic analysis of data obtained at any temperature program, T(t). The method has been used successfully for a large variety of thermally stimulated processes [1,2,4], including those that occur on cooling, i.e., crystallization [28,29,30], gelation [31,32,33], and solid-solid transition [34]. It affords evaluating *E_α_* as a function of conversion α, by finding a minimum the function:

(14) $$\Psi \left(E_{\alpha }\right)=\overset{n}{\underset{i=1}{\sum }}\overset{n}{\underset{j\neq i}{\sum }}\frac{J\left[E_{\alpha },T_{i}\left(t_{\alpha }\right)\right]}{J\left[E_{\alpha },T_{j}\left(t_{\alpha }\right)\right]}$$

where:

(15) $$J\left[E_{\alpha },T_{i}\left(t_{\alpha }\right)\right]\equiv \int_{t_{\alpha -\Delta \alpha }}^{t_{\alpha }}\exp\left[\frac{-E_{\alpha }}{RT_{i}\left(t\right)}\right]dt$$

and *n* is the number of the temperature programs. It is important to note that this method belongs to the class of flexible [4,35] integral methods, i.e., the methods in which the user can control the limits of integration. This is essential for isoconversional calculations on cooling data because some of the most popular methods (e.g., Ozawa, Flynn and Wall, Starink, etc) [26] are rigid integral methods. They are applicable exclusively to the heating data. The issue is discussed in detail elsewhere [4,30,35]. As an alterative to the rigid integral methods one can also use differential isoconversional methods.

In addition to the *E_α_* vs α dependences we also evaluated the *E_α_* vs T dependences. The latter is readily evaluated by replacing each value of *α* with the mean value of the temperatures, *T_α_* related to this *α* at different cooling rates. 

The preexponential factor, *A_α_* as a function of conversion was determined by substituting the *E_α_* values into the equation of the compensation effect [26]:

(16) $$\ln A_{\alpha }=a+bE_{\alpha }$$

The parameters *a* and *b* were estimated via fitting the pairs of *E_i_* and ln*A_i_* into Equation (16). The *E_i_* and ln*A_i_* values were found by substituting the reaction models, *f_i_*(*α*), into the linear form of the basic rate equation [26]:

(17) $$\ln\left(\frac{d\alpha }{dt}\right)-\ln\left[f_{i}\left(\alpha \right)\right]=\ln A_{i}-\frac{E_{i}}{RT}$$

For each *f_i_*(*α*), the *E_i_* and ln*A_i_* values were evaluated respectively from the slope and intercept of the linear plot of the left-hand side of Equation (17) vs the reciprocal temperature. As determined previously [18], four *f*(*α*) functions that represent the power law (P2, P3, P4) and Avrami-Erofeev (A2) models [26] are sufficient for obtaining accurate values of the preexponential factor. Here we must stress the need of using a differential method for evaluating the *E_i_* and ln*A_i_* values. Recall, that all our calculations are done on cooling data. However, most popular model-fitting methods (e.g., Coats-Redfern) [26] are rigid integral methods and, as stated earlier, inapplicable to cooling data.

Finally, we used the values of *E_α_* and ln*A_α_* to determine the rate constant. It has been done by substituting the respective values into the Arrhenius Equation (8) so that for each given temperature *T_α_* the rate constant is determined as [36]:

(18) $$\ln k=\ln A_{\alpha }-\frac{E_{\alpha }}{RT_{\alpha }}$$

## 7. Representative Examples

**Figure 3:**
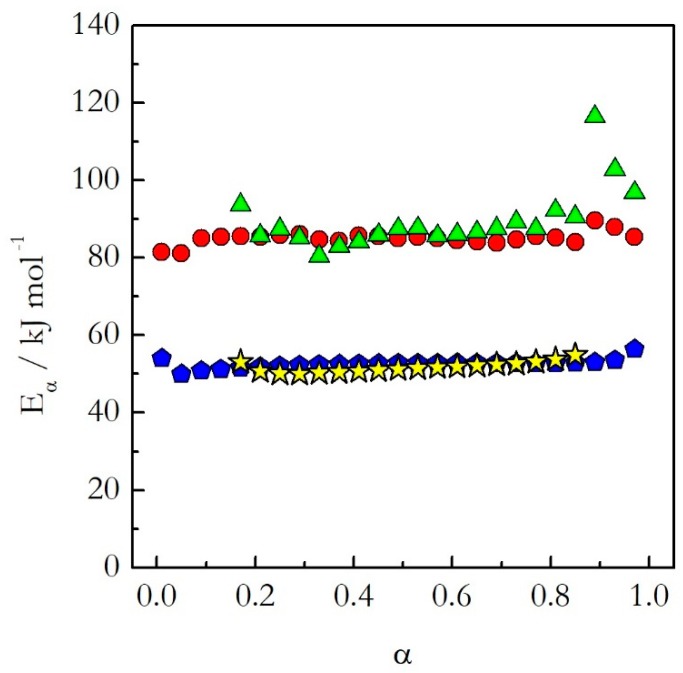
The activation energy as a function of conversion for the thermal decomposition of AN (heating (red circles) and cooling (green triangles)) and nonstoichiometric polymerization of DGEBA and m-PDA (heating (blue pentagons) and cooling (yellow stars)) evaluated on heating and cooling [20,22]. Partially adapted from Ref. [20] with permission from the PCCP Owner Societies.

**Figure 4:**
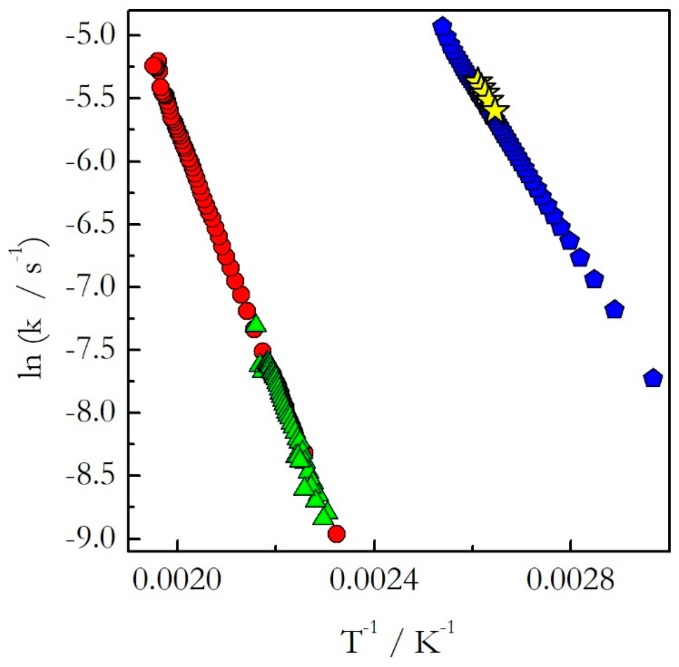
The rate constant as a function of reciprocal temperature for the thermal decomposition of AN (heating (red circles) and cooling (green triangles)) and nonstoichiometric polymerization of DGEBA and m-PDA (heating (blue pentagons) and cooling (yellow stars)) evaluated on heating and cooling. Adapted from Ref. [23] with permission from Elsevier.

Having covered the practical aspects of the data analysis, we can now return to the central hypothesis of the kinetic studies on cooling. As already stated, for single-step reactions the kinetics on heating and cooling are expected to be nearly identical, whereas for multi-step reactions significantly different. The single-step reactions demonstrate activation energies that do not vary with conversion. Figure 3 shows isoconversional activation energy evaluated for the thermal decomposition of ammonium nitrate (AN) [20] and nonstoichiometric polymerization of diglycidyl ether of bisphenol A (DGEBA) epoxy and *m*-phenylenediamine (*m*-PDA) [22]. Both reactions are examples of a single-step process. As seen in Figure 3, for these processes the activation energy evaluated on heating agrees well with that determined on cooling. Similar results have been obtained for the preexponential factor [20,22]. The similarity of the Arrhenius parameters for heating and cooling translates naturally into similar Arrhenius plots (Figure 4). One can see that for the considered reactions of decomposition and polymerization the Arrhenius plots estimated by Equation (18) for heating and cooling experiments practically coincide with each other. 

This means that as long as the reaction is single-step, one should not expect any significant difference in the kinetics determined on heating and cooling. In other words, if a reaction exhibits constant activation energy one can use the Arrhenius parameters evaluated on heating to predict the kinetic behavior on cooling. 

On the other hand, multi-step reactions typically exhibit the activation energy that varies with conversion. As illustrated above by using a purely algebraic example, one may expect significant differences in the kinetics measured on heating and cooling when the activation energy depends on both temperature and conversion. A representative example of such reaction is crosslinking polymerization of DGEBA with m-PDA. This type of reactions tends to manifest a transition from a kinetic to a diffusion regime so that their overall kinetics is described by rate equations that include the reaction and diffusion rate constants [4]. The respective rate equation proposed by Vyazovkin and Sbirrazzuoli yields the following form of the effective activation energy [37]:

(19) $$E=\frac{E_{D}k\left(T\right)+Ek_{D}\left(T,\alpha \right)}{k\left(T\right)+k_{D}\left(T,\alpha \right)}$$

where *E_D_* and *E* are the activation energies of diffusion and reaction, *k*(*T*) is the reaction rate constant, and *k_D_*(*T*,*α*) is the diffusion rate constant. The latter depends not only on temperature but also on conversion because diffusion slows down with increasing the extent of polymerization. Therefore, the activation energy for crosslinking polymerization is generally a function of both conversion and temperature.

**Figure 5:**
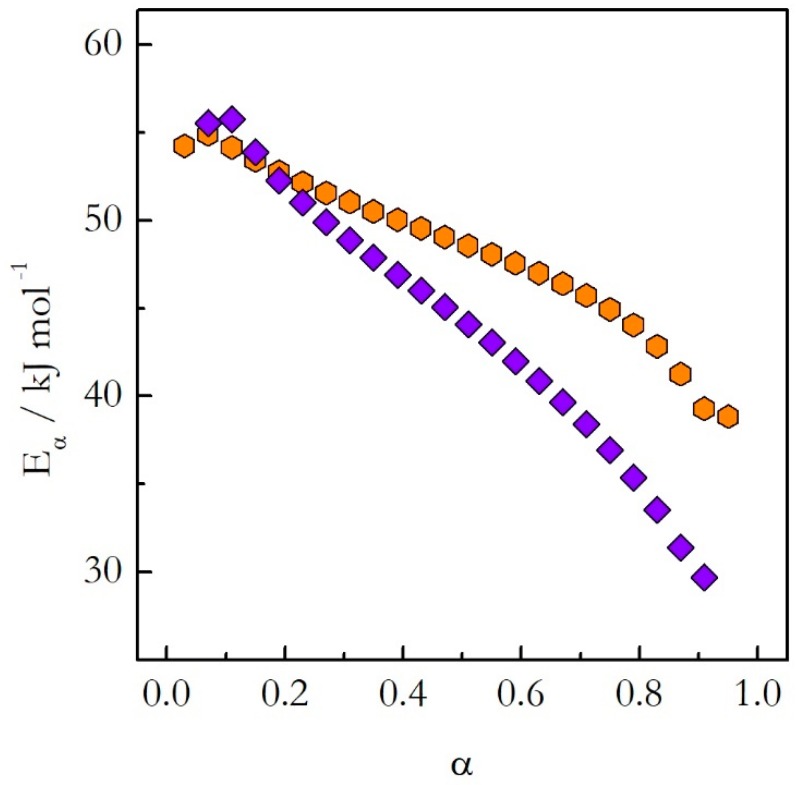
The activation energy as a function of conversion for the stoichiometric polymerization of DGEBA and m-PDA evaluated on heating (orange hexagons) and cooling (violet diamonds) [22,23]. Adapted from Ref. [22] with permission from Wiley.

**Figure 6:**
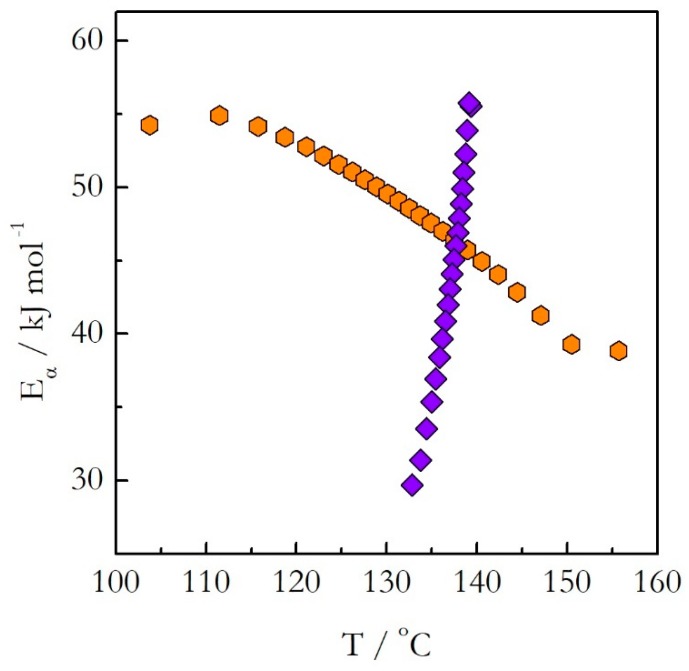
The isoconversional activation energy as a function of temperature for the stoichiometric polymerization of DGEBA and m-PDA evaluated on heating (orange hexagons) and cooling (violet diamonds) [22,23]. Adapted from Ref. [22] with permission from Wiley.

We have studied the crosslinking polymerization of DGEBA with m-PDA on heating and on cooling from 140 °C [22,23]. The application of the advanced isoconversional method to the obtained data has yielded the dependences of *E_α_* on α presented in Figure 5. The activation energy decreases with the reaction progress on both heating and cooling. However, the values do not match each other. A more dramatic difference is revealed when the activation energy is plotted against temperature as shown in Figure 6. The *E_α_* on T dependences evaluated on heating and cooling exhibit the opposite trends. The activation energy decreases with increasing temperature on heating and increases with increasing temperature on cooling. 

**Figure 7:**
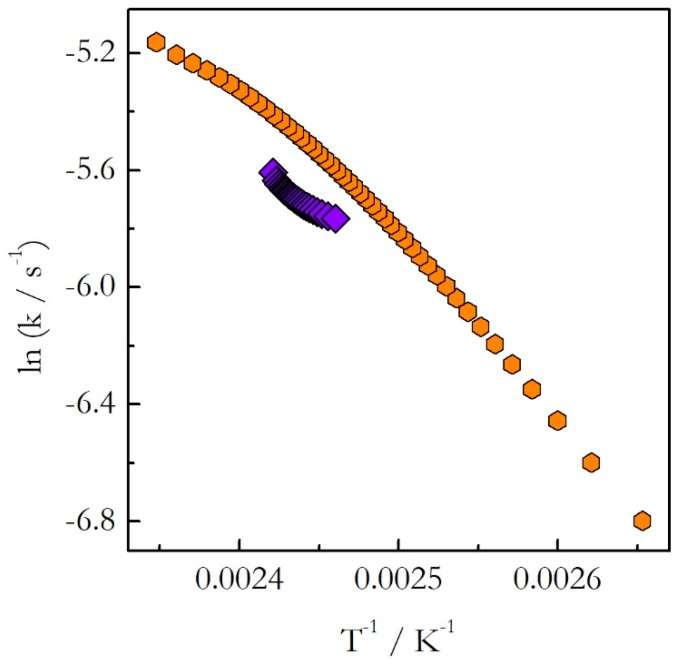
The rate constant as a function of reciprocal temperature for the stoichiometric polymerization of DGEBA and m-PDA evaluated on heating (orange hexagons) and cooling (violet diamonds) [23]. Adapted from Ref. [23] with permission from Elsevier.

As explained earlier, the opposite trends in E with respect to temperature mean the opposite curvatures of the respective Arrhenius plots. The experimental Arrhenius plots determined by Equation (18) are shown in Figure 7. 

Indeed, the plot obtained for heating is concave down (E decreases with T), whereas for cooling it is concave up (E increases with T). Obviously, the lines of the opposite curvatures cannot coincide. This means that the kinetics evaluated on heating cannot be used to predict the kinetic behavior during cooling. This example confirms clearly our argument that generally one should not assume that the kinetics measured on heating should be similar to that measured on cooling. This assumption holds true for single-step reactions, i.e., reactions for which the activation energy does not vary with conversion. If the activation energy is found to vary with conversion, one should avoid extrapolating heating kinetics to the cooling conditions. Instead, the cooling kinetics should be studied on their own to determine adequate kinetic parameters. 

## 8. Conclusions

In the real world, many thermally stimulated reactions occur during continuous cooling. However, the kinetics of thermally stimulated reactions are customarily studied under continuous heating conditions. The relevance of such studies to the cooling conditions rests upon the assumption that the kinetics on cooling are the same as on heating. Our studies demonstrate that this holds only for single-step reactions, whereas for multi-step reactions the difference between the respective kinetics can be very significant. This highlights the practical need in continuing systematic studies of thermally stimulated reactions occurring during continuous cooling. Conducting such studies presents a number of challenges not encountered in routine kinetic studies on heating. This overview article has shared our experience in addressing these challenges. Together with the other issues discussed, this paper is expected to provide sufficient background knowledge to help other workers to have a quick and successful start in their kinetic studies of thermally stimulated reactions that occur during continuous cooling.
